# The use of out-of-hours primary care during the first year of the COVID-19 pandemic

**DOI:** 10.1186/s12913-022-08096-x

**Published:** 2022-05-21

**Authors:** Lotte Ramerman, Corinne Rijpkema, Nanne Bos, Linda E. Flinterman, Robert A. Verheij

**Affiliations:** 1 Nivel, Netherlands Institute for Health Services Research, Postbus 1568, Utrecht, 3500 BN The Netherlands; 2 Tranzo, Tilburg University, Postbus 90153, Tilburg, 5000 LE the Netherlands

**Keywords:** After-hours care, Primary health care, COVID-19, Health services, Electronic health records

## Abstract

**Background:**

In the Netherlands, General Practitioners (GP) are usually the first point of contact with a health professional for most health problems. Out-of-hours (OOH) primary care is provided by regional OOH services. Changes in consultation rates at OOH services may be regarded as a warning system for failures elsewhere in the healthcare system. Therefore in this study, we investigated how the COVID-19 pandemic changed the use of primary care OOH services during the first year of the pandemic.

**Methods:**

Routine electronic health records data were used from 60% of OOH services in the Netherlands, collected by the Nivel Primary Care Database. We compared consultation rates per week (2020) for COVID-19-like symptoms and other health problems (e.g. small traumas, urinary tract infections), for different age groups, the proportion of remote consultations, and different levels of urgency during the pandemic compared to the same period in 2019.

**Results:**

The number of consultations for COVID-19-like symptoms peaked at the start of the COVID-19 pandemic, while consultations for other health problems decreased. These changes in consultation rates differed between age groups. Remote consultations took place more frequently for all health problems, while the proportion of non-urgent health problems increased.

**Conclusion:**

There were significant changes in the number of consultations and the proportion that were remote for COVID-19-like symptoms and other health problems. Especially care for babies and young children decreased, while the number of consultations for older adults remained stable. The continued use of OOH services by older adults suggests there were unmet care needs elsewhere in our healthcare system.

**Supplementary Information:**

The online version contains supplementary material available at 10.1186/s12913-022-08096-x.

## Background

In the Netherlands, General Practitioners (GPs) serve as the first point of contact when patients experience health problems. Therefore, GPs are the gatekeepers to more specialized (hospital) care [1, 2]. Out-of-hours (OOH) services provide GP care outside office hours [3]. When patients need immediate care, which cannot wait until the next day when their own GP is available, patients consult an OOH service [3]. In addition to more acute health problems presented at OOH services, such as traumata or infections, also patients with health problems related to chronic conditions are frequent users of OOH services [4]. When usual care for the management of chronic conditions is disrupted during the daytime, exacerbations of the condition of these patients may occur. If such exacerbations occur outside of office hours, patients consult OOH services [4, 5]. Therefore, OOH services may be considered a safety net of our healthcare system and can serve as an indicator for adverse effects of changes elsewhere in our healthcare system [6].

The COVID-19 pandemic has had an enormous impact on health systems [7], including the organization of primary care [8]. During office hours, face-to-face consultations decreased in GP care, while remote consultations increased [9–12]. Self-care among patients with chronic conditions was advocated, disincentivizing routine check-ups with their GPs [13]. Furthermore, Homeniuk and Collins found that frequent attenders in GP practices- babies, young children, and older adults- had considerably fewer consultations during office hours for non-COVID-19-related symptoms in the first phase of the pandemic [9]. To avoid infections, patient flows were separated [14], patients avoided care for fear of COVID-19 infection [10, 15, 16], and patients were discouraged to visit the GP’s premises.

Until now, little is known about how the containment measures and the pandemic itself affected the use of OOH services. Jansen et al. (2020) [6] suggested that the consequences of unmet care needs elsewhere in our health system due to the pandemic may be detected by monitoring the use of OOH care. Furthermore, OOH services can help fill the gap for overcrowded regular care caused by increasing COVID-19 infections [6]. A similar suggestion was made in the UK; using OOH services for COVID-19-related care, when hospitalization is not required, preferably provided remotely [17]. First results from Belgium showed a temporary increase in contacts with OOH services, where almost half of all consultations were COVID-19-related [18]. However, OOH services also implemented measures limiting the use of OOH services and to prevent the spread of the coronavirus among patients and healthcare professionals. Therefore, a different approach for providing and organizing OOH care was warranted, similar to GP care during office hours. This included discouraging patients to visit an OOH location and further implementation of remote consultations (for example by phone, video, or digital) [18, 19].

This study aimed to assess the impact of the COVID-19 pandemic on OOH services, in terms of healthcare use and how care was provided. We analyzed changes in the use of care from OOH services, overall and for different ages of patients, during the first year of the pandemic (2020) for COVID-19-like symptoms and other health problems. Furthermore, we analyzed changes in the use of remote consultations versus physical consultations.

## Methods

### Design and data source/study population

We used deidentified routinely recorded electronic health records data from OOH services, from 2019 and 2020, who participated in Nivel Primary Care Database (Nivel-PCD). Data from 27 (2019) and 32 (2020) OOH services and all their locations were included in the analyses, representing a joint catchment area of almost 12 million people in both years. The included OOH services represented 60% of OOH services in the Netherlands and approximately 70% of its population. The data used were representative of the Dutch population concerning sex, age, and distribution of regions [20].

### Privacy and ethics

Ethics approval for this study was waived by the medical ethics committee of the University Medical Centre Groningen (reference number: 2020/309). Obtaining informed consent from patients or approval by a medical ethics committee is not obligatory for observational studies using electronic health records when the database does not contain directly identifiable data (art. 24 GDPR Implementation Act jo art. 9.2 sub j GDPR). Furthermore, this study was approved according to the governance code of Nivel-PCD under the number NZR-00320.087.

### Outcome measures

The primary outcome of the study was the use of care from OOH services, defined by the number of consultations with OOH services per 100.000 inhabitants in the catchment area (consultation rate), including remote consultations (for example by phone), consultations at OOH locations or home visits. We distinguished between consultations for COVID-19-like symptoms and consultations for other health problems.

There were no data available on confirmed diagnosis for COVID-19, therefore, we established a list of COVID-19-like symptoms (based on International Classification of Primary Care 1; ICPC1 codes) to assess consultation rates in 2019 and 2020, to evaluate the likely increase of consultations for these symptoms due to the COVID-19 pandemic [21]. ICPC1-codes for COVID-19-like symptoms included acute upper respiratory infection (R74), other respiratory infections (R83), pneumonia (R81), other virus infections (A77), other infectious diseases (A78), fever (A03), shortness of breath (R02), coughing (R05) or influenza (R80). Consultations for all other diagnoses were regarded as unrelated to COVID-19 infections (other health problems, e.g. small traumas, urinary tract infections). GPs assigned diagnoses by ICPC1-coding during the consultation with an OOH service. GPs may record multiple diagnoses for each consultation/patient, but most record one (97.3%). If more than one diagnosis was recorded, consultations were considered for COVID-19-like symptoms, if any of the diagnoses were for one of the above-mentioned ICPC1-codes.

Secondary outcomes included the use of OOH services for different age categories, the proportion of remote consultations, and the urgency level assigned to the consultation by triage. We used the following age categories: 0-4 years, 5-17 years, 18-44 years, 45-69 years, and ≥ 70 years. Remote consultations were mostly provided by phone, often by the triagist, under the supervision of a GP. Furthermore, each consultation with an OOH service was preceded by telephone triage in which an urgency level was assigned using a standardized set of criteria (Nederlandse Triage Standaard) [22], ranging from U0 (loss of vital functions; immediate care) to U5 (minor health problem; patient can visit own GP during office hours).

### Phases of the COVID-19 pandemic in the Netherlands

To evaluate the changes in the use of care at OOH services during the COVID-19 pandemic, the different phases of the pandemic in the Netherlands in 2020 should be considered in the analyses, based on weekly infection rates [23] and related national containment measures (Table 1) [24].

**Table 1:** Different phases of the COVID-19 pandemic in the Netherlands, related to infection rates and containment measures

Phase of pandemic	Period in 2020	Description of phase
**Phase 0**	weeks 0-8	Period before the first case of COVID-19, healthcare as usual
**Phase 1**	weeks 9-24	Period with the first wave of infections starting after the first case of COVID-19 in the Netherlands. Measures included social distancing (keeping 1.5 m distance), closing schools, restaurants, and sports facilities, and working from home (Intelligent lockdown).
**Phase 2**	weeks 25-37	Period of fewer COVID-19 infections during summer. More limited measures, including social distancing.
**Phase 3**	weeks 38-52	The second wave of COVID-19 infections. Measures included social distancing, closing schools, restaurants, sports facilities, non-essential stores, working from home, wearing face masks (hard lockdown).

### Data analyses

The characteristics of the patient population of the OOH services in 2019 and 2020, were described by the size of the catchment area, the total number of consultations and number of individual patients overall and per age group, and sex. Relative changes in consultation rates in 2020 compared to 2019 were plotted for each week. Consultations of patients with COVID-19-like symptoms and for other health problems were presented and analyzed separately. Differences between 2019 and 2020 in consultation rates were analyzed using linear regression with an interaction between phases of the COVID-19 pandemic and year, to establish the effect on consultation rates during the different phases of the COVID-19 pandemic, overall and for different age groups. Standard errors were corrected for the auto-correlation in the time series. Changes in the proportion of remote consultations were analyzed using logistic regression with an interaction between phases and year, and with corrected standard errors for the auto-correlation in the time series. Differences between 2019 and 2020 in the proportion of the different urgency levels and the proportion of the reasons for consultation (by ICPC chapter) were analyzed using a two-proportion z-test. Analyzes were performed using a significance threshold of 0.05 and using STATA SE 16.

## Results

In 2020, OOH services were consulted at least once by 14.8% of the population of the joint catchment area of the OOH services, compared to 16.3% in 2019 (Table 2).

**Table 2:** Characteristics of OOH services in 2019 and 2020

	2019	2020
**Total population of joint catchment area**	11,970,895	12,068,119
**Number of contacts**	3,015,476	2,791,426
**Number of contacts per 1000 inhabitants**	251	231
**Number of patients with contacts (%)**	1,948,658 (16.3%)	1,785,644 (14.8%)
**Age**		
0-4 years (contacts per 1000 pop.)	695	546
5-17 years (per 1000 pop)	219	183
18-44 years (per 1000 pop.)	225	213
45-69 years (per 1000 pop.)	173	174
70 years and older (per 1000 people)	369	371
**Sex**		
Male (%)	49.6	47.2
Female (%)	50.4	52.8

### The overall use of OOH services

**Fig. 1:**
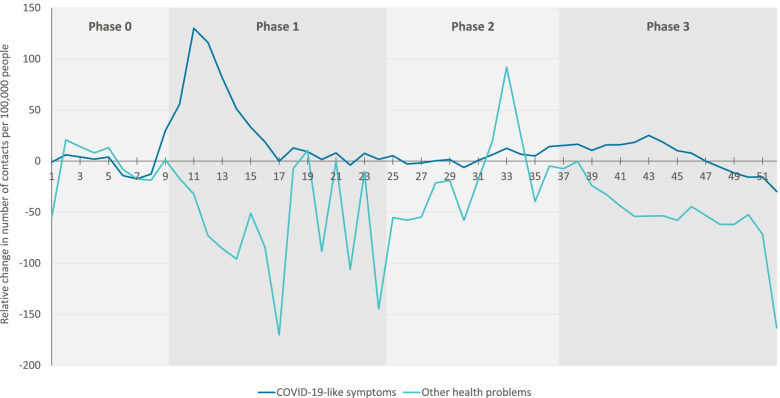
Relative consultation rates in 2020 compared to 2019 (per 100.000 inhabitants) from OOH services during the COVID-19 pandemic. The relative use of care is separately presented for patients with COVID-19-like symptoms and patients with other health problems unrelated to COVID-19

Consultation rates for COVID-19-like symptoms increased rapidly and temporarily after the outbreak of COVID-19 infections in the Netherlands (Phase 1) (Fig. 1). However, the increase was followed by a drop in the number of consultations, therefore, mean consultation rates during this phase did not differ significantly from 2019 (Supplementary file 1). After the peak of consultations for COVID-19-like symptoms in phase 1, consultations for other health problems unrelated to COVID-19 dropped significantly (*p* < 0.001); up to 25% fewer consultations per week at the OOH services, than the same period in 2019 (Fig. 1; Additional file 1). During a calmer period (Phase 2) with fewer infections, consultation rates were back to normal: there were no significant differences in the use of OOH services for both consultations for COVID-19-like symptoms and other health problems compared to 2019 (respectively *p* = 0.119 and *p* = 0.133). From September (Phase 3), the number of infections increased again, and concurrently, the number of consultations for other health problems decreased significantly (*p* = 0.006); up to 15% fewer consultations than in 2019 at OOH services. The consultations for COVID-19-like symptoms did not increase significantly (*p* = 0.685) during phase 3, compared to the same period in 2019.

Starting phase 1 of the COVID-19 pandemic, we observed small, but significant changes in the health problems for which OOH services were consulted, based on different body systems according on the ICPC main chapters, compared to the same period in 2019 (Supplementary file 2).

### Use of OOH services in different age groups

**Fig. 2:**
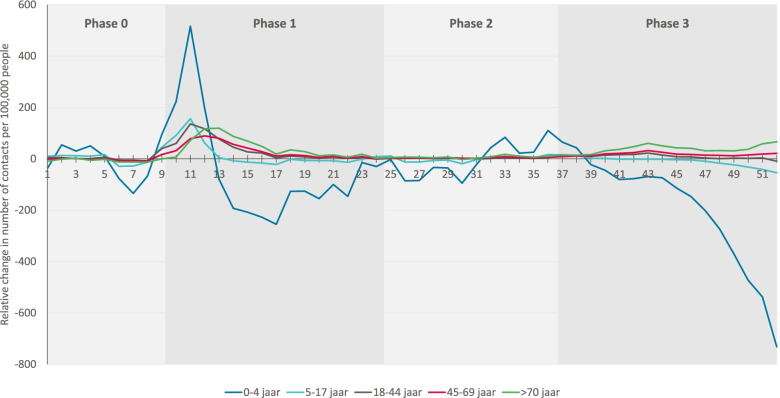
Relative consultation rates in 2020 compared to 2019 per 100.000 inhabitants from OOH services during the COVID-19 pandemic for COVID-19-like symptoms, by age group

Consultation rates for adult patients (≥18 years) related to COVID-19-like symptoms increased significantly (*p* < 0.02), starting phase 1, compared to 2019 (Fig. 2). In contrast, the number of consultations for COVID-19-like symptoms for babies and young children (0-4 years) did not increase on average during phases 1 and 2. Furthermore, during phase 3, there were considerably fewer consultations for this group of symptoms for patients aged 0-4 years (*p* = 0.006), than in the same period in 2019.

**Fig. 3:**
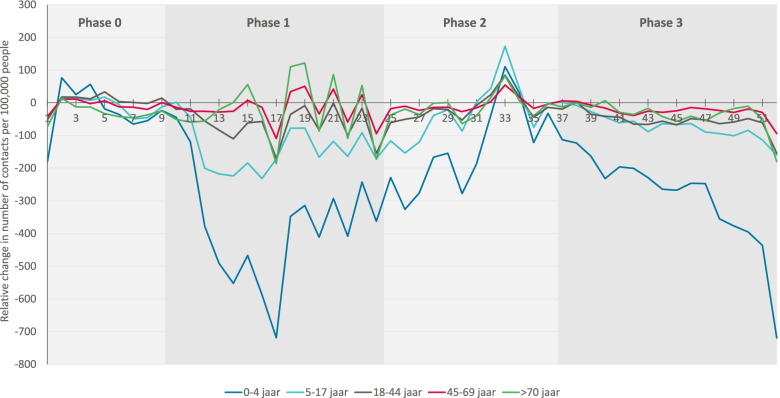
Relative consultation rates in 2020 compared to 2019 per 100.000 inhabitants from OOH services during the COVID-19 pandemic for other health problems unrelated to covid-19, by age

Figure 3 illustrates that the effects of the COVID-19 pandemic on consultation rates for other health problems unrelated to COVID-19 differed between age categories. On average, patients aged 0-4 years showed a significant decline in the number of consultations with OOH services during phase 1 (*p* < 0.001), up to 50% fewer consultations, after which their use of care remained lower during phases 2 and 3 (*p* < 0.001), compared to 2019. Patients aged 5-44 years also showed a significant decrease during phases 1 and 3 (*p* < 0.002), up to 32-50% fewer consultations. During phase 2, a calmer period with fewer infections, consultation rates were not significantly lower than in 2019. Consultation rates for other health problems in patients aged ≥45, did not differ between 2020 and 2019.

### Use of remote consultations

**Fig. 4:**
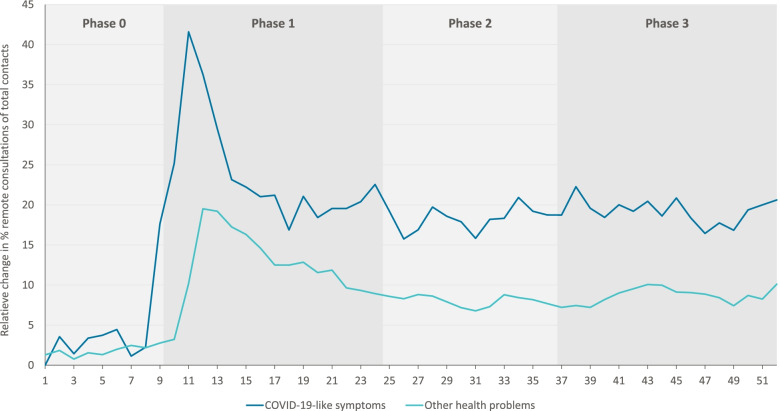
Percent-points change in proportion of remote consultations in 2020 compared to 2019 for COVID-19-like symptoms and other health problems

At the start of the COVID-19 pandemic (Phase 1), a steep increase was seen in the proportion of remote consultations (by telephone or digital) both for COVID-19-like symptoms (*p* < 0.02; up to 42%-point increase) and for other health problems (*p* > 0.02; up to 23%-point increase), compared to 2019 (Fig. 4). Concurrently, the proportion of consultations at an OOH location decreased, while the proportion of home visits for COVID-19-like symptoms increased slightly and remained stable for other health problems. Although the percentage of remote consultations decreased again later in phase 1, they remained higher than in 2019. From September 2020 (Phase 3), the increasing infection rates during this period did not coincide with the second increase in remote consultations.

### Urgency levels of the provided OOH services

Assigned urgency levels changed significantly (Table 3) in the period after the start of the COVID-19 pandemic (phases 1-3), compared to the same period in 2019. The percentage of non-urgent health problems (U4/5) increased, while the percentage of more urgent health problems (U2/3) decreased. In addition, the proportion of contacts for highly urgent health problems increased slightly.

**Table 3:** Urgency level of consultations with ooh services; percentage of total number of consultations measured for the start of the COVID-19 pandemic in The Netherlands (Phase 1, March 2020), compared with the same period in 2019

Urgency level assigned to consultations^**a**^	2019	2020	Z (*p*-value)**
U1 (%)	2.6	2.8	−18.07 (*p* < 0.001)
U2 (%)	16.6	15.4	37.07 (*p* < 0.001)
U3 (%)	39.1	35.4	86.35 (*p* < 0.001)
U4 (%)	13.8	14.6	−23.11 (*p* < 0.001)
U5 (%)	27.9	32.9	− 96.89 (*p* < 0.001)

^a^U0 (loss of vital functions; immediate care) to U5 (minor health problem; patient can visit own GP during office hours). U0 was not assigned and excluded from the table

**Differences between 2019 and 2020 (starting phase 1) in the proportion of the different urgency levels were analyzed using a z-test

## Discussion

Only at the beginning of the COVID-19 pandemic, an increase was observed in consultations for COVID-19-like symptoms, likely due to fear and uncertainty concerning COVID-19. A decrease in consultation rates for other health problems unrelated to COVID-19, followed the peak in consultations for COVID-19-like symptoms, after which the consultation rates remained consistently lower than in 2019, except for the summer months in which the virus was less active. Furthermore, there was a slight shift in the health problems presented at OOH services. There are several possible explanations for the decrease in consultations rates for these other health problems: fear of contamination at healthcare locations, the overall belief that GPs and other health professionals were overloaded [8, 15, 18, 25], and patients were asked to avoid care in general when possible. Zhang (2020) argued that fear for contamination, especially among vulnerable people, had severe consequences: more avoided care and consequently more deaths [15]. A similar decline in consultation for these other health problems as observed in this study was observed in Belgium, while consultations with OOH services for patients at risk for COVID-19 were much higher [18]. This might be related to differences between these countries in the organization of primary (COVID-19) care and the availability of COVID-testing facilities.

Changes in the use of OOH services differed between age groups. Babies and young children were frequent users of OOH services. However, after an initial peak at the start of the pandemic, their overall use of care declined considerably for other health problems. A similar effect was found in GP care during office hours [9]. During the second wave of COVID-19 infections in the Netherlands (Phase 3), babies and young children had fewer consultations for COVID-19-like symptoms than in 2019, such as respiratory infections, fever, and coughing. The lower use of care is likely to be associated with policy measures that were taken to prevent the spread of the virus (i.e. lockdown measures), including the closing of daycare facilities, schools, social distancing, and a general decline in social activity. This may have eliminated potential sources of infection, not only for COVID-19 but for all respiratory infections, which are often the reason to consult the OOH services for young children. A similar pattern was observed for the yearly epidemic of respiratory syncytial virus (RSV) in young children. Circulation of RSV halted after the introduction of containment measures for COVID-19 [26].

In contrast, consultation rates for older adults (≥45 years) remained at a similar level as in 2019 for consultations for health problems unrelated to COVID-19. In GP care during office hours a decrease in consultations was found for patients aged 70 years and older [9]. For OOH services we did not observe the same effect. Despite the overall decrease in the use of OOH services, this did not occur for older adults, indicating the continued and possibly increased need for acute care by these patients. This might be related to avoided or delayed care elsewhere in our healthcare system. Routine check-ups by GPs and medical specialists were canceled or delayed, increasing the risk of exacerbations [5]. Moreover, there was an increase in consultations for COVID-19-like symptoms, which was in line with the overall higher infection rates, more severe illness, and higher mortality rates in older adults [27–29].

As a result of the COVID-19 pandemic, more consultations were remote, by phone, video, or digital. The triage before consultation with OOH services was more strict, applying a higher threshold for healthcare. Therefore, high urgency levels were assigned less quickly and more consultations were remote. The study of Morreel et al. (2020) also showed an increase in remote consultations in OOH care [18], however, the increase of remote consultations seemed even more prominent in the Netherlands. The implementation of, and the experiences gained, using remote consultations may benefit OOH services in the care they provide after the pandemic, keeping in mind the long-term health outcomes when using remote consultations. Some claim that the care provision became more efficient, leaving more time for consultations with patients with more severe complaints [7]. Others claim that symptoms of more serious illness might be missed and that remote consultation will lead to less person-centered care [18, 19]. Therefore, the long-term effects of this increase in remote consultations should be monitored.

During the period studied, the continued use of OOH care by older adults, while the use decreased among other ages, may suggest an effect of avoided or delayed care in other parts of the healthcare system on OOH services. Further research is necessary to study the underlying mechanisms explaining the use of OOH services in relation to decreased GP care during the day and specialized care as a result of the COVID-19 pandemic. The current study does show that the reduction in the use of OOH services was mainly for younger patients. Further analyses are necessary to provide more insight into the use of care of these specific patient groups who are frequent users of OOH services, such as older adults, people with chronic conditions or babies, and young children.

### Strengths and limitations

A strength of this study was the use of routinely recorded healthcare data from about two-thirds of the Netherlands, encompassing a joint population of 12 million individuals, representative of the whole country. A limitation of the study was the lack of data on confirmed diagnosis for COVID-19 and limited means to validate the selection of ICPC1-codes that we used to identify consultations for COVID-19-like symptoms, which was theory-driven. We provided an estimate by selecting consultations for COVID-19-like symptoms in 2020 and comparing the healthcare for these health problems with the same period in 2019. However, while interpreting the results, one should consider that COVID-19 did not only add to the regular number of consultations for these symptoms but also replaced them.

## Conclusions

In conclusion, this study shows clear changes in the use of OOH services during the COVID-19 pandemic, both in the number of consultations and how the care was provided. Especially babies and young children showed a strongly decreased use of OOH services, which coincided with the closing of daycare and schools, eliminating a likely source of different kinds of infections. The continued use of OOH care for older adults, while other age groups showed a decreased use of OOH care, suggests that delayed or avoided care does affect OOH services, while more in-depth analyses are necessary to better understand the underlying mechanisms. A better understanding of the impact of the COVID-19 pandemic on OOH services within the context of our entire healthcare system is essential for the future organization of OOH services and the preparedness for future pandemics. The results presented here may serve as a baseline for the effects of avoided or delayed care later on, during, and after the pandemic and as a starting point for further analysis of different patient groups.

## Supplementary Information

**Additional file 1.**

**Additional file 2.**

## Data Availability

The data underlying this article will be shared at reasonable request to the corresponding author, following the governance of the ‘Nivel Primary Care Database’. Data in the ‘Nivel Primary Care Database’ are extracted from the electronic health records of OOH services. The use of the data for research purposes is subject to approval by a committee representing the health professionals who recorded the data in their electronic health record, reviewing proposals on the relevance for, and privacy of, the OOH services and their patients. (https://www.nivel.nl/en/nivel-zorgregistraties-eerste-lijn/nivel-primary-care-database).

## Abbreviations

COVID-19: Coronavirus Disease 2019
GP: General Practitioner
ICPC: International Classification of Primary Care
OOH services: Out-of-hours services
