# Supplementary material for: The use of out-of-hours primary care during the first year of the COVID-19 pandemic
Source: BMC Health Serv Res. 2022 May 21;22:679. doi: 10.1186/s12913-022-08096-x (PMC9122805; doi:10.1186/s12913-022-08096-x)
Supplement: Supplementary file 1 — Additional file 1. [file 12913_2022_8096_MOESM1_ESM.docx]

**Supplementary file 1. Consultation rates (per 100.000 inhabitants) and the proportion of remote consultations from OOH services for each of the phases of the COVID-19 pandemic, compared to the same period in 2019. The use of care is separately presented for patients with COVID-19-like symptoms and patients with other health problems unrelated to COVID-19.**

|  |  | **2019** | | **2020** | |  |  |  |
| --- | --- | --- | --- | --- | --- | --- | --- | --- |
|  |  | Mean | SD | Mean | SD | Coef. | CI 95% | P-value |
| **Overall** |  |  |  |  |  |  |  |  |
| COVID-19-like symptoms | Phase 0 | 59 | 10,5 | 55 | 5,2 | -3.84 | -13.69 6.02 | 0.445 |
|  | Phase 1 | 39 | 8,6 | 66 | 46,2 | 27.77 | -3.26 58.80 | 0.079 |
|  | Phase 2 | 29 | 4,5 | 32 | 4,4 | 3.20 | -0.83 7.24 | 0.119 |
|  | Phase 3 | 46 | 18,3 | 48 | 9,2 | 2.68 | -10.29 15.66 | 0.685 |
| Other health problems | Phase 0 | 415 | 27.7 | 407 | 7,4 | -7.46 | -26.03 11.12 | 0.432 |
|  | Phase 1 | 459 | 46.4 | 394 | 41,3 | -65.68 | -100.56 -30.80 | <0.001 |
|  | Phase 2 | 445 | 29.8 | 427 | 23,7 | -18.56 | -42.77 5.65 | 0.133 |
|  | Phase 3 | 421 | 46.1 | 371 | 50,1 | -50.49 | -86.21 -14.78 | 0.006 |
| **0-4 years of age** |  |  |  |  |  |  |  |  |
| COVID-19-like symptoms | Phase 0 | 511 | 68,9 | 490 | 36,1 | -21.46 | -83.47 40.55 | 0.498 |
|  | Phase 1 | 342 | 68,0 | 302 | 235,6 | -39.60 | -199.79 120.60 | 0.628 |
|  | Phase 2 | 254 | 53,7 | 253 | 41,3 | -0.80 | -45.62 44.02 | 0.972 |
|  | Phase 3 | 490 | 222,3 | 283 | 23,0 | -206.78 | -355.03 -58.53 | 0.006 |
| Other health problems | Phase 0 | 898 | 72.0 | 873 | 49,0 | -24.61 | -90.49 41.28 | 0.464 |
|  | Phase 1 | 997 | 95.6 | 637 | 133,1 | -360.06 | -458.95 -261.18 | <0.001 |
|  | Phase 2 | 874 | 87.9 | 738 | 52,1 | -137.14 | -204.75 -69.53 | <0.001 |
|  | Phase 3 | 983 | 171.8 | 701 | 88,2 | -281.54 | -398.65 -164.44 | <0.001 |
| **5-17 years of age** |  |  |  |  |  |  |  |  |
| COVID-19-like symptoms | Phase 0 | 69 | 28,4 | 68 | 17,0 | -1.08 | -29.57 27.42 | 0.941 |
|  | Phase 1 | 34 | 12,1 | 51 | 56,3 | 16.80 | -20.61 54.22 | 0.379 |
|  | Phase 2 | 24 | 8,8 | 24 | 7,7 | -0.04 | -7.89 7.81 | 0.992 |
|  | Phase 3 | 32 | 16,5 | 21 | 4,1 | -11.40 | -22.68 -0.13 | 0.047 |
| Other health problems | Phase 0 | 350 | 32.6 | 339 | 27,8 | -11.91 | -47.50 23.69 | 0.512 |
|  | Phase 1 | 426 | 55.3 | 292 | 56,4 | -134.48 | -182.47 -86.49 | <0.001 |
|  | Phase 2 | 404 | 74.2 | 377 | 36,0 | -26.77 | -81.61 28.07 | 0.339 |
|  | Phase 3 | 340 | 21.2 | 270 | 39,8 | -70.03 | -97.02 -43.03 | <0.001 |
| **18-44 years of age** |  |  |  |  |  |  |  |  |
| COVID-19-like symptoms | Phase 0 | 26 | 5,6 | 26 | 4,5 | -0.14 | -5.92 5.65 | 0.963 |
|  | Phase 1 | 18 | 4,3 | 53 | 44,4 | 35.23 | 5.83 64.62 | 0.019 |
|  | Phase 2 | 14 | 1,2 | 17 | 2,5 | 2.81 | 1.19 4.43 | 0.001 |
|  | Phase 3 | 18 | 6,3 | 27 | 5,9 | 8.86 | 3.62 14.10 | 0.001 |
| Other health problems | Phase 0 | 380 | 32,0 | 384 | 10,7 | 3.83 | -18.18 25.83 | 0733 |
|  | Phase 1 | 429 | 48,6 | 369 | 45,0 | -60.09 | -98.66 -21.51 | 0.002 |
|  | Phase 2 | 433 | 25,5 | 418 | 26,0 | -15.80 | -38.73 7.13 | 0.177 |
|  | Phase 3 | 399 | 39,0 | 349 | 46,7 | -49.77 | -81.58 -17.96 | 0.002 |
| **45-69 years of age** |  |  |  |  |  |  |  |  |
| COVID-19-like symptoms | Phase 0 | 22 | 3,9 | 19 | 2,6 | -3.04 | -6.69 0.61 | 0.102 |
|  | Phase 1 | 15 | 3,9 | 46 | 31,0 | 30.41 | 9.71 51.10 | 0.004 |
|  | Phase 2 | 11 | 0,9 | 15 | 1,8 | 4.38 | 3.22 5.54 | <0.001 |
|  | Phase 3 | 15 | 5,4 | 34 | 10,9 | 18.99 | 11.94 26.04 | <0.001 |
| Other health problems | Phase 0 | 302 | 23,0 | 294 | 9,3 | -7.84 | -24.28 8.60 | 0.350 |
|  | Phase 1 | 329 | 38,3 | 312 | 34,8 | -17.05 | -45.90 11.81 | 0.247 |
|  | Phase 2 | 329 | 16,5 | 324 | 16,6 | -4.89 | -20.12 10.34 | 0.529 |
|  | Phase 3 | 304 | 37,6 | 284 | 43,3 | -20.47 | -50.25 9.32 | 0.178 |
| **≥70 years of age** |  |  |  |  |  |  |  |  |
| COVID-19-like symptoms | Phase 0 | 53 | 9,5 | 46 | 6,9 | -6.88 | -15.37 1.62 | 0.112 |
|  | Phase 1 | 41 | 7,9 | 82 | 41,4 | 40.91 | 13.46 68.37 | 0.003 |
|  | Phase 2 | 31 | 3,3 | 39 | 3,7 | 7.44 | 4.50 10.37 | <0.001 |
|  | Phase 3 | 39 | 10,6 | 81 | 28,7 | 41.58 | 23.59 59.57 | <0.001 |
| Other health problems | Phase 0 | 678 | 42,1 | 648 | 28,0 | -30.39 | -65.66 4.89 | 0.091 |
|  | Phase 1 | 698 | 65,0 | 674 | 61,1 | -23.88 | -69.28 21.52 | 0.303 |
|  | Phase 2 | 652 | 31,8 | 641 | 30,3 | -11.40 | -38.92 16.12 | 0.417 |
|  | Phase 3 | 651 | 92,6 | 627 | 108,1 | -24.13 | -99.40 51.15 | 0.530 |
| **The proportion of remote consultations** |  |  |  |  |  |  |  |  |
| COVID-19-like symptoms | Phase 0 | 36,7% | 1,6% | 39,5% | 2,7% | 0.12 | 0.0161013 0.218424 | 0.023 |
|  | Phase 1 | 38,3% | 1,0% | 55,6% | 8,5% | 0.70 | 0.4825494 0.9229763 | <0.001 |
|  | Phase 2 | 40,0% | 1,8% | 56,7% | 1,3% | 0.67 | 0.6238742 0.7235931 | <0.001 |
|  | Phase 3 | 37,7% | 0,7% | 54,9% | 1,3% | 0.70 | 0.6634732 0.7307867 | <0.001 |
| Other health problems | Phase 0 | 44,9% | 0,5% | 46,7% | 1,1% | 0.07 | 0.0413121 0.1047697 | <0.001 |
|  | Phase 1 | 44,6% | 0,5% | 55,9% | 4,2% | 0.45 | 0.3472965 0.5596432 | <0.001 |
|  | Phase 2 | 45,5% | 0,6% | 53,2% | 0,6% | 0.31 | 0.2862405 0.3289149 | <0.001 |
|  | Phase 3 | 45,5% | 0,5% | 53,9% | 1,0% | 0.34 | 0.309618 0.3674361 | <0.001 |
