# Supplementary material for: The use of out-of-hours primary care during the first year of the COVID-19 pandemic
Source: BMC Health Serv Res. 2022 May 21;22:679. doi: 10.1186/s12913-022-08096-x (PMC9122805; doi:10.1186/s12913-022-08096-x)
Supplement: Supplementary file 2 — Additional file 2. [file 12913_2022_8096_MOESM2_ESM.docx]

****Supplementary file 2**** Reason for consultation WITH OOH SERVICES per ICPC main chapter; PERCENTAGE OF TOTAL NUMBER OF CONSULTATIONS MEASURED FOR THE START OF THE COVID-19 PANDEMIC IN THE NETHERLANDS (PHASE 1, MARCH 2020), COMPARED WITH THE SAME PERIOD IN 2019.

| **ICPC main chapter** | **2019** | **2020** | **Z (p-value)*** |
| --- | --- | --- | --- |
| A General | 14.82% | 16.84% | -63.43 (p<0.001) |
| B Blood | 0.41% | 0.41% | -0.78 (p=0.435) |
| D Digestive tract | 12.65% | 11.83% | 26.39 (p<0.001) |
| F Eye | 3.28% | 3.06% | 12.98 (p<0.001) |
| H Ear | 3.42% | 2.43% | 64.57 (p<0.001) |
| K Cardiovascular | 4.17% | 4.63% | -25.85 (p<0.001) |
| L Musculoskeletal system | 17.30% | 16.57% | 19.72 (p<0.001) |
| N Nervous system | 3.83% | 3.97% | -8.97 (p<0.001) |
| P Mental | 3.44% | 3.89% | -27.46 (p<0.001) |
| R Respiratory tract | 10.93% | 12.80% | -65.84 (p<0.001) |
| S Skin | 13.88% | 13.51% | 10.50 (p<0.001) |
| T Endocrine/metabolic | 1.60% | 1.55% | 3.24 (p=0.001) |
| U Urinary tract | 5.47% | 5.79% | -16.21 (p<0.001) |
| W Pregnancy/labor | 0.53% | 0.54% | -1.45 (p=0.148) |
| X Reproductive system Women | 0.88% | 0.89% | -2.04 (p=0.042) |
| Y Reproductive system Men | 0.62% | 0.63% | -0.09 (p=0.387) |
| Z Social problems | 0.26% | 0.29% | -6.25 (p<0.001) |

* Differences between 2019 and 2020 (starting phase 1) in the proportion of the different urgency levels were analyzed using a z-test.
